# Noninvasive assessment of fibrosis among patients with nonalcoholic fatty liver disease [NAFLD]

**DOI:** 10.1016/j.metop.2021.100158

**Published:** 2022-01-05

**Authors:** David Bernstein, Alexander J. Kovalic

**Affiliations:** Department of Internal Medicine, Division of Hepatology, Donald and Barbara Zucker School of Medicine at Hofstra/Northwell Health, North Shore University Hospital, Hempstead, NY, USA

## Abstract

Nonalcoholic fatty liver disease [NAFLD] is a condition affecting a vast portion of the worldwide population. The presence of underlying fibrosis is the strongest predictor of long-term outcomes and mortality, with a graduated increase in liver-related morbidity and mortality with progression from moderate fibrosis tobiomarkers targeting collagen turnover and extracellular matrix remodeling FibroTest FAST™, Velacur™, MRE]. While many of these provide a robust, stand alone value, the accuracy of these noninvasive tests markedly increase when used in combination or in sequential order with one another. There is not a uniform consensus demonstrating superiority of any specific test. Given the growing role and accuracy of these tests, they should have an expanding role in the assessment of fibrosis across this patient population and obviate the need for liver biopsy in a large portion of patients. Future clinical studies should focus on validating these novel biomarkers, as well as optimizing the sequential or algorithmic testing when combining these noninvasive tests.

## 1 Introduction

Nonalcoholic fatty liver disease [NAFLD] constitutes a wide spectrum of pathology defined by the presence of hepatic steatosis. Recently, the prevalence of NAFLD has been estimated to affect approximately one-quarter of the global population [1]. In the United States alone, this prevalence translates to over 80 million individuals with the diagnosis of NAFLD [2]. Given the vast number of patients affected by this disease process, it may seem unsurprising that NAFLD alone has already been shown to place a considerable amount of economic burden and stress on health care systems of European countries [3] as well as the United States [4]. Similarly, the prevalence of NAFLD in China is also currently on the rise [5]. Fueled by the inexhaustible obesity epidemic, rampant prevalence of metabolic syndrome, in tandem with lack of targeted treatment options, the breadth and impact of NAFLD will continue to expand over the years to come.

Moving down the spectrum from simple isolated hepatic steatosis, the pathophysiology of NAFLD portends a natural progression, triggered by lipotoxicity, towards hepatocyte injury and fibrosis. The first step incites the lipotoxicity necessary for the diagnosis of nonalcoholic steatohepatitis [NASH]. Approximately one-quarter of patients with NAFLD will progress to NASH [6]. Approximately one-quarter of patients with NASH have at least F2 fibrosis at the time of their diagnosis, and furthermore, one-quarter of patients with NASH will go on to develop cirrhosis [F4] [6]. As the progression towards the more severe end of the NAFLD spectrum continues, patients are at higher risk for complications of cirrhosis, extrahepatic manifestations and hepatocellular carcinoma [HCC]. The annual incidence of HCC is estimated at 1–2% among patients with NASH cirrhosis [7]. Among candidates for liver transplantation, NASH has become one of the primary culprits for the rise in HCC incidence in the United States [8].

The gold standard for diagnosis and staging of NAFLD is liver biopsy, defined by a minimum of 5% hepatocyte steatosis. Liver biopsy provides an assessment of hepatic steatosis, inflammation and fibrosis but is limited by sample size and non-equal distribution of these findings throughout the liver. Liver biopsy is currently the only modality to identify the presence of nonalcoholic steatohepatitis [NASH] [9]. However, given its widespread prevalence, pursuing a liver biopsy for every patient with NAFLD is not a pragmatic clinical approach. Limitations of liver biopsy include pain, infection, bleeding, pneumothorax, limited sample variation, and not to mention additional time and resources. Determination of the degree of hepatic fibrosis is the most important factor in determining the risks associated with NASH progression. Therefore, there is significant interest and research towards the development of simple, reproducible non-invasive tests to assess the degree of in NAFLD, which will be the primary focus of this review.

## 2 Importance and clinical impact of fibrosis assessment in NAFLD

It is important to delineate the terms revolving around fibrosis in the current literature. Significant fibrosis is defined as a minimum stage fibrosis of two or more [F ≥ 2]. Advanced fibrosis is either stage three or four [F ≥ 3]. Stage four fibrosis [F4] is designated as cirrhosis. The significance of defining and detecting hepatic fibrosis among patients with NAFLD lies within its clinical implications and mortality.

Among patients with NAFLD, it has been repeatedly demonstrated that progression of the degree of fibrosis is the strongest predictor of long-term outcomes and mortality [[10], [11], [12], [13]]. One meta-analysis demonstrated a progression of increased mortality associated with significant fibrosis, advanced fibrosis, and cirrhosis [12]. This meta-analysis included 1495 biopsy-proven NAFLD patients across 17,452 patient-years of follow-up investigating the liver-related and all-cause mortality. Overall, the liver-related mortality displayed a stepwise relationship to fibrosis stage, with mortality rate ratio [MRR] of patients with F1 1.41 [95% CI 0.17–11.95], F2 9.57 [95% CI 1.67–54.93], F3 16.69 [2.92–95.36], and F4 42.30 [95% CI 3.51–510.34]. Similarly, all-cause mortality among patients with F1, F2, F3, and F4 stage fibrosis demonstrated a MRR of 1.58 [95% CI 1.19–2.11], 2.52 [95% CI 1.85–3.42], 3.48 [95% CI 2.51–4.83], and 6.40 [95% CI 4.11–9.95], respectively. This landmark meta-analysis clearly illustrates the graduated increase in liver-related and all-cause mortality as the fibrosis stage advances along the NAFLD spectrum.

Another study sought out to identify the most important factors for hepatic decompensation among NAFLD patients using a combination of factors. Among an international cohort of 299 biopsy-proven NAFLD patients [only included patients with cirrhosis], the ABIDE model [consisting of AST/ALT ratio, total bilirubin, INR, history of type 2 diabetes mellitus, and presence of esophageal varices] was tested using a derivation and validation cohort [14]. Using a threshold for the ABIDE model of ≥4.1, it was established that these patients have significantly higher risk of decompensating events [hazard ratio 6.7; 95% CI 4.0–11.2; p < 0.001], five year cumulative incidence [37% versus 6%, p < 0.001], and shorter duration to decompensating events [3.8 versus 6.7 years; p < 0.001]. ABIDE model performed with significantly greater accuracy and AUROC as compared to NFS, FIB-4, MELD, and CTP scoring systems. While no direct analysis was made for fibrosis via histology, the ABIDE model demonstrated robust diagnostic accuracy and clinical utility. However, this study was limited to biopsy-proven NAFLD and cirrhosis. The external validity to all patients on the entire spectrum of NAFLD remain to be validated. The ABIDE model may be particularly limiting given that one of its components includes the presence or absence of esophageal varices. The evaluation of varices with endoscopy is likely a missing piece of clinical information among the majority of patients with isolated hepatic steatosis, and without signs of cirrhosis. Further analysis of the ABIDE model and similar scoring systems will need to be validated across a greater spectrum of NAFLD patients among future studies.

While new studies may guide future models predicting hepatic decompensation using noninvasive modalities, the presence of underlying fibrosis currently remains the benchmark for predicting future liver-related events and mortality.

## 3 Noninvasive serum biomarkers based on conventional testing

There have been a number of noninvasive modalities proposed to risk stratify patients with NAFLD in order to systematically assess for fibrosis. These noninvasive serum biomarkers are fully delineated in Table 1 based on the components of each test. Common thresholds utilized in the fibrosis assessment among NAFLD are illustrated in Table 2.

**Table 1:** Noninvasive serum biomarkers and their individual components.

Conventional-based tests			Tests based upon collagen turnover and extracellular matrix remodeling		
Name of Test	Abbreviation	Components	Name of Test	Abbreviation	Components
AST to Platelet Ratio Index	APRI	Platelets AST	Enhanced Liver Fibrosis test	ELF	Hyaluronic acid Amino-terminal propeptide of type III procollagen Tissue inhibitor of metallopeptidase-1
Fibrosis-4 Index	FIB-4	Age Platelets ALT AST	FibroTest [FibroSure in the USA]	–	Bilirubin GGT Haptoglobin Apolipoprotein A1 Alpha-2 macroglobulin
			Hepascore	–	Age Gender Bilirubin GGT Hyaluronic acid Alpha-2 macroglobulin
NAFLD Fibrosis Score	NFS	Age BMI History of diabetes mellitus [or impaired fasting glucose] Platelets Albumin ALT AST	ADAPT	–	Age History of diabetes mellitus Amino-terminal propeptide of type III procollagen Platelets
			FibroMeter V2G	–	Age Gender BUN Platelets INR AST alpha-2 macroglobulin Hyaluronic acid
Hepamet Fibrosis Score	HFS	Age Gender History of diabetes mellitus Platelets Albumin AST HOMA	FibroMeter V3G	–	Age Gender BUN Platelets INR AST GGT alpha-2 macroglobulin
			NIS4	–	miR-34a-5p Alpha-2 macroglobulin YKL-40 Glycated hemoglobin

Abbreviations: AST, aspartate aminotransferase; ALT, alanine aminotransferase; BMI, body mass index; HOMA, homeostatic model assessment for insulin resistance; BUN, blood urea nitrogen; GGT, gamma-glutamyl transferase; INR, international normalized ratio; YKL-40, Chitinase-3-like protein 1.

**Table 2:** Scoring table for common thresholds utilized for noninvasive testing.

	High risk for advanced fibrosis	Intermediate risk	Likely excludes advanced fibrosis
APRI	>1.5	0.5–1.5	<0.5
FIB-4	<1.3	1.3–2.67	>2.67
NFS	>0.675	−1.455–0.675	< −1.455
HFS	>0.47	0.47–0.12	<0.12
ELF	>10.35	n/a	<10.35
FibroTest/FibroSure	>0.58	n/a	<0.58
NIS4	>0.63	0.63–0.36	<0.36
FAST	>0.67^a^	0.67–0.35^a^	<0.35^a^

a Numbers reported here are with respect to significant fibrosis among patients with NASH.

### 3.1 AST to platelet index

The AST to platelet index [APRI] was first established among patients with chronic hepatitis C in order to detect significant fibrosis and even cirrhosis [15]. APRI does not require a special laboratory and can be easily calculated by the practitioner utilizing readily available on-line calculators. The specific formula for APRI is equal to 100 x (AST/upper limit of normal)/platelet count. Most of the clinical trials among NAFLD patients have compared APRI to other noninvasive testing modalities that will be further detailed below in addition to Table 3.

**Table 3:** High impact clinical studies reporting diagnostic accuracy, sensitivity, specificity, and AUROC for conventional-based noninvasive tests as compared to liver biopsy for the diagnosis of significant fibrosis, advanced fibrosis, and cirrhosis.

Study	Study Design	Country	Number of biopsy-proven NAFLD patients	Name of test	Threshold	Sensitivity			Specificity			AUROC		
						Significant Fibrosis	Advanced Fibrosis	Cirrhosis	Significant Fibrosis	Advanced Fibrosis	Cirrhosis	Significant Fibrosis	Advanced Fibrosis	Cirrhosis
Younes et al., 2021 [1]	Cross-sectional	Spain, Italy, United Kingdom	1173											
				APRI	n/a	54.2%	70.2%	71.4%	75.5%	66.0%	64.4%	0.669	0.720	0.709
				BARD	n/a	80.5%	55.0%	65.7%	42.0%	69.8%	66.3%	0.651	0.677	0.736
				FIB-4	n/a	54.9%	58.8%	80.0%	73.7%	80.3%	79.1%	0.697	0.733	0.856
				NFS	n/a	57.8%	84.7%	88.6%	74.9%	55.6%	80.6%	0.700	0.761	0.876
				HFS	n/a	71.5%	87.0%	91.4%	69.8%	61.4%	67.9%	0.758	0.805	0.820
Drolz et al., 2021 [2]	Retrospective	Germany	386	AST/ALT	n/a	n/a	n/a	n/a	n/a	n/a	n/a	n/a	0.710	n/a
				APRI	0.29	n/a	81.0%	n/a	n/a	72.0%	n/a	n/a	0.848	n/a
				BARD	n/a	n/a	n/a	n/a	n/a	n/a	n/a	n/a	0.708	n/a
				FIB-4	1.0	n/a	88.0%	n/a	n/a	80.0%	n/a	n/a	0.904	n/a
				NFS	−0.4	n/a	79.0%	n/a	n/a	64.0%	n/a	n/a	0.750	n/a
Castellana et al., 2021 [3]	Meta-analysis	Multinational	12,604											
				FIB-4	<1.3	n/a	76.0%	n/a	n/a	67.0%	n/a	n/a	n/a	n/a
				FIB-4	>2.67	n/a	39.0%	n/a	n/a	95.0%	n/a	n/a	n/a	n/a
				FIB-4	<1.3 and >2.67	n/a	65.0%	n/a	n/a	93.0%	n/a	n/a	n/a	n/a
				NFS	<-1.455	n/a	81.0%	n/a	n/a	64.0%	n/a	n/a	n/a	n/a
				NFS	>0.676	n/a	34.0%	n/a	n/a	94.0%	n/a	n/a	n/a	n/a
				NFS	<-1.455 and >0.676	n/a	61.0%	n/a	n/a	93.0%	n/a	n/a	n/a	n/a
Shah et al., 2009 [4]	Retrospective	USA	541											
				AST/ALT		n/a	n/a	n/a	n/a	n/a	n/a	n/a	0.742	n/a
				APRI		n/a	n/a	n/a	n/a	n/a	n/a	n/a	0.720	n/a
				BARD score		n/a	n/a	n/a	n/a	n/a	n/a	n/a	0.700	n/a
				FIB-4		n/a	n/a	n/a	n/a	n/a	n/a	n/a	0.802	n/a
				NFS		n/a	n/a	n/a	n/a	n/a	n/a	n/a	0.768	n/a
Angulo et al., 2007 [5]	Prospective	Multinational	733											
				NFS	>0.676	n/a	43.0%	n/a	n/a	96.0%	n/a	n/a	0.820	n/a
				NFS	<-1.455	n/a	77.0%	n/a	n/a	71.0%	n/a	n/a	0.820	n/a

### 3.2 Fibrosis-4 index

The Fibrosis-4 Index [FIB-4] test is a noninvasive test initially created from a panel for staging liver disease among patients with HCV alone [16], as well as HCV and HIV co-infection [17]. However, FIB-4 was subsequently validated among patients with NAFLD. The FIB-4 score is calculated by the following formula: (age x AST)/(platelets x √ALT). FIB-4 does not require a special laboratory and can be easily calculated by the practitioner utilizing readily available on-line calculators.

An early retrospective validation study cemented FIB-4 as a robust noninvasive test in the evaluation of advanced fibrosis in NAFLD [18]. Tested across 541 biopsy-proven patients with NAFLD, FIB-4 demonstrated significantly superior diagnostic accuracy than AST/ALT ratio, APRI, BARD score, Goteborg University Cirrhosis Index, and cirrhosis discriminant score. FIB-4 did not outperform NFS, however, with both tests achieving similar diagnostic accuracy for detection of advanced fibrosis with AUROC 0.802 and 0.768, respectively.

Overall, the thresholds that are most validated include a lower limit of 1.3 and upper limit of 2.67 for FIB-4 testing [Table 2, Table 3, Table 4, Table 5, Table 6]. The lower threshold of 1.3 effectively rules out advanced fibrosis with 82% sensitivity, while using an upper limit of 2.67 serves as an effect surrogate marker for diagnosing advanced fibrosis with 96% specificity.

**Table 4:** High impact clinical studies reporting diagnostic accuracy, sensitivity, specificity, and AUROC for noninvasive tests targeting collagen turnover and extracellular matrix remodeling as compared to liver biopsy for the diagnosis of significant fibrosis, advanced fibrosis, and cirrhosis.

Study	Study Design	Country	Number of biopsy-proven NAFLD patients	Name of test	Threshold	Sensitivity			Specificity			AUROC		
						Significant Fibrosis	Advanced Fibrosis	Cirrhosis	Significant Fibrosis	Advanced Fibrosis	Cirrhosis	Significant Fibrosis	Advanced Fibrosis	Cirrhosis
Harrison et al., 2020 [6]	Prospective	Multinational	239											
				NIS4	<0.36	n/a	81.5%	n/a	n/a	63.0%	n/a	n/a	0.800	n/a
				NIS4	>0.63	n/a	50.7%	n/a	n/a	87.1%	n/a	n/a	0.800	n/a
Daniels et al., 2019 [7]	Retrospective	Multinational	431											
				APRI		n/a	n/a	n/a	n/a	n/a	n/a	n/a	0.730	n/a
				FIB-4		n/a	n/a	n/a	n/a	n/a	n/a	n/a	0.780	n/a
				NFS		n/a	n/a	n/a	n/a	n/a	n/a	n/a	0.780	n/a
				PRO-C3		n/a	n/a	n/a	n/a	n/a	n/a	n/a	0.810	n/a
				ADAPT	6.3287	n/a	90.9%	n/a	n/a	72.7%	n/a	n/a	0.860	n/a
Loomba et al., 2019 [8]	Retrospective	USA	396											
				Alpha-2 macroglobulin		n/a	n/a	n/a	n/a	n/a	n/a	n/a	0.772	n/a
				Hyaluronic acid		n/a	n/a	n/a	n/a	n/a	n/a	n/a	0.812	n/a
				TIMP metallopeptidase inhibitor 1		n/a	n/a	n/a	n/a	n/a	n/a	n/a	0.782	n/a
				Combination of three above	17	n/a	84.8%	n/a	n/a	72.3%	n/a	n/a	0.867	n/a
				FIB-4		n/a	n/a	n/a	n/a	n/a	n/a	n/a	0.774	n/a
				NFS		n/a	n/a	n/a	n/a	n/a	n/a	n/a	0.610	n/a
Staufer et al., 2019 [9]	Prospective	Austria	186											
				FIB-4	2.67	38.0%	49.0%	54.0%	97.0%	96.0%	93.0%	0.800	0.820	0.860
				NFS	n/a	n/a	n/a	n/a	n/a	n/a	n/a	0.780	0.800	0.790
				ELF	9.8	82.0%	72.0%	78.0%	76.0%	90.0%	85.0%	0.850	0.900	0.920
				FibroMeter V2G	0.309	80.0%	81.0%	88.0%	80.0%	81.0%	78.0%	0.860	0.880	0.950
				FibroMeter V3G	0.378	78.0%	84.0%	88.0%	80.0%	78.0%	75.0%	0.840	0.880	0.940
				VCTE	8.2 kPa	83.0%	92.0%	90.0%	68.0%	77.0%	73.0%	0.850	0.910	0.950
Boursier et al., 2016 [10]	Cross-sectional	France	452											
				BARD	2	n/a	79.1%	n/a	n/a	50.7%	n/a	0.698	0.695	0.694
				NFS	−1.036	n/a	76.7%	n/a	n/a	60.0%	n/a	0.717	0.732	0.766
				APRI	0.559	n/a	61.0%	n/a	n/a	76.4%	n/a	0.719	0.754	0.767
				FIB-4	1.515	n/a	75.6%	n/a	n/a	67.1%	n/a	0.721	0.780	0.777
				FibroTest	0.316	n/a	81.4%	n/a	n/a	56.8%	n/a	0.716	0.736	0.761
				Hepascore	0.322	n/a	67.4%	n/a	n/a	76.1%	n/a	0.753	0.778	0.807
				FibroMeter [V2G]	0.453	n/a	76.7%	n/a	n/a	71.8%	n/a	0.786	0.817	0.824
				VCTE	8.7 kPa	n/a	88.4%	n/a	n/a	62.9%	n/a	0.842	0.831	0.864
Guha et al., 2008 [11]	Retrospective	United Kingdom	192											
				NFS		n/a	n/a	n/a	n/a	n/a	n/a	0.860	0.890	n/a
				ELF		n/a	n/a	n/a	n/a	n/a	n/a	0.900	0.930	n/a
				NFS and ELF		n/a	n/a	n/a	n/a	n/a	n/a	0.930	0.980	n/a

**Table 5:** High impact clinical studies reporting diagnostic accuracy, sensitivity, specificity, and AUROC for noninvasive imaging as compared to liver biopsy for the diagnosis of significant fibrosis, advanced fibrosis, and cirrhosis.

Study	Study Design	Country	Number of biopsy-proven NAFLD patients	Name of test	Threshold	Sensitivity			Specificity			AUROC		
						Significant Fibrosis	Advanced Fibrosis	Cirrhosis	Significant Fibrosis	Advanced Fibrosis	Cirrhosis	Significant Fibrosis	Advanced Fibrosis	Cirrhosis
Troelstra et al., 2021 [12]	Prospective	Netherlands	37	MRE	2.30 kPa	n/a	100.0%	n/a	n/a	78.6%	n/a	n/a	0.920	n/a
				VCTE [FibroScan]	9.9 kPa	n/a	87.5%	n/a	n/a	69.0%	n/a	n/a	0.770	n/a
Qu et al., 2021 [13]	Prospective	China	237											
				VCTE [FibroTouch]	9.4 kPa	58.0%	68.0%	80.0%	82.0%	72.0%	71.0%	0.710	0.710	0.770
Newsome et al., 2020 [14]	Prospective	Multinational	350											
				FAST	<0.35	n/a	90.0%	n/a	n/a	53.0%	n/a	n/a	0.800	n/a
				FAST	>0.67	n/a	48.0%	n/a	n/a	90.0%	n/a	n/a	0.800	n/a
Siddiqui et al., 2019 [15]	Prospective	USA	393											
				VCTE	8.6 kPa	n/a	80.0%	n/a	n/a	74.0%	n/a	n/a	0.830	n/a
				VCTE	13.1 kPa	n/a	n/a	89.0%	n/a	n/a	86.0%	n/a	n/a	0.930
Chen et al., 2017 [16]	Prospective	Multinational	111											
				VCTE	7.6 kPa	82.1%	84.2%	81.8%	77.6%	63.8%	92.4%	0.830	0.840	0.900
				MRE	4.52 kPa	82.1%	84.2%	81.8%	89.8%	82.8%	90.9%	0.930	0.920	0.950
Xiao et al., 2017 [17]	Meta-analysis	Multinational	13,046											
				APRI	0.43–1.5	59.3%	72.9%	56.2%	77.1%	67.7%	83.6%	0.700	0.750	0.750
				BARD	2	44.3%	83.0%	52.2%	70.4%	59.0%	83.8%	0.640	0.730	0.700
				FIB-4	0.37–2.67	64.4%	77.8%	76.4%	70.0%	95.7%	82.4%	0.750	0.800	0.850
				NFS	−1.455	65.5%	72.9%	80.0%	82.5%	73.8%	80.8%	0.720	0.780	0.830
				VCTE [FibroScan M probe]	n/a	91.7%	88.9%	96.5%	57.4%	66.3%	77.7%	0.830	0.870	0.920
				VCTE [Fibroscan XL probe]	n/a	75.8%	75.3%	87.8%	64.8%	74.0%	82.0%	0.820	0.860	0.940
				SWE	2.67–10.6	85.0%	89.9%	100.0%	94.4%	91.8%	85.6%	0.890	0.910	0.970
				MRE	3.4–6.7	73.2%	85.7%	86.6%	90.7%	90.8%	93.4%	0.880	0.930	0.920
Park et al., 2017 [18]	Prospective	USA	104											
				VCTE	6.9 kPa	79.3%	77.8%	63.5%	84.6%	77.6%	66.3%	0.860	0.800	0.690
				MRE	2.9 kPa	79.3%	77.8%	75.0%	81.8%	80.3%	81.4%	0.890	0.870	0.870
				VCTE	7.9 kPa	n/a	90.0%	n/a	n/a	65.0%	n/a	n/a	0.860	n/a
					9.6 kPa	n/a	74.0%	n/a	n/a	81.0%	n/a	n/a	0.860	n/a
Petta et al., 2017 [19]	Retrospective	Multinational	324											
				FIB-4	n/a	n/a	n/a	n/a	n/a	n/a	n/a	n/a	0.792	n/a
				NFS	n/a	n/a	n/a	n/a	n/a	n/a	n/a	n/a	0.774	n/a
				VCTE	n/a	n/a	n/a	n/a	n/a	n/a	n/a	n/a	0.863	n/a
Imajo et al., 2016 [20]	Prospective	Japan	142											
				VCTE	7–14 kPa	65.2%	85.7%	100.0%	88.7%	83.8%	75.9%	0.820	0.880	0.920
				MRE	2.5–6.7 kPa	87.3%	74.5%	90.9%	85.0%	86.9%	94.5%	0.890	0.890	0.970
Tapper et al., 2016 [21]	Prospective	USA	164											
				NFS	n/a	n/a	n/a	n/a	n/a	n/a	n/a	n/a	0.770	n/a
				VCTE	9.9 kPa	n/a	95.0%	n/a	n/a	77.0%	n/a	n/a	0.930	n/a
Cassinotto et al., 2016 [22]	Prospective	France	291											
				ARFI	0.95–2.04	n/a	n/a	n/a	n/a	n/a	n/a	0.770	0.840	0.840
				VCTE	6.2–16.1 kPa	n/a	n/a	n/a	n/a	n/a	n/a	0.820	0.860	0.870
				SWE	6.3–14.4 kPa	n/a	n/a	n/a	n/a	n/a	n/a	0.860	0.890	0.880
Petta et al., 2015 [23]	Retrospective	Italy	179											
				VCTE	7.9–9.6 kPa	n/a	85.0%	n/a	n/a	81.0%	n/a	n/a	0.860	n/a

**Table 6:** High impact clinical studies reporting diagnostic accuracy, sensitivity, specificity, and AUROC for combination and sequential noninvasive testing as compared to liver biopsy for the diagnosis of significant fibrosis, advanced fibrosis, and cirrhosis.

Study	Study Design	Country	Number of biopsy-proven NAFLD patients	Name of test	Threshold	Sensitivity			Specificity			AUROC		
						Significant Fibrosis	Advanced Fibrosis	Cirrhosis	Significant Fibrosis	Advanced Fibrosis	Cirrhosis	Significant Fibrosis	Advanced Fibrosis	Cirrhosis
Mózes et al., 2021 [24]	Meta-analysis	Multinational	5735											
				AST/ALT	0.64	n/a	75.0%	n/a	n/a	47.0%	n/a	n/a	0.640	n/a
				APRI	0.49	n/a	67.0%	n/a	n/a	63.0%	n/a	n/a	0.700	n/a
				FIB-4	1.44	n/a	69.0%	n/a	n/a	70.0%	n/a	n/a	0.760	n/a
				NFS	−1.39	n/a	75.0%	n/a	n/a	63.0%	n/a	n/a	0.730	n/a
				VCTE	9.1	n/a	77.0%	n/a	n/a	78.0%	n/a	n/a	0.850	n/a
				FIB-4/VCTE^a^	<0.88 and ≥2.31/<7.4 and ≥12.1 kPa	n/a	80.0%	n/a	n/a	81.0%	n/a	n/a	n/a	n/a
				FIB-4/VCTE^a^	<1.3 and ≥2.67/<7.9 and ≥9.6 kPa	n/a	67.0%	n/a	n/a	85.0%	n/a	n/a	n/a	n/a
				FIB-4/VCTE^a^	<1.3 and ≥2.67/<8.0 and ≥10.0 kPa	n/a	66.0%	n/a	n/a	86.0%	n/a	n/a	n/a	n/a
				NFS/VCTE^a^	<-2.55 and ≥0.28/<7.4 and ≥12.1 kPa	n/a	77.0%	n/a	n/a	83.0%	n/a	n/a	n/a	n/a
				NFS/VCTE^a^	<-1.455 and ≥0.676/<7.9 and ≥9.6 kPa	n/a	65.0%	n/a	n/a	86.0%	n/a	n/a	n/a	n/a
				NFS/VCTE^a^	<-1.455 and ≥0.676/<8.0 and ≥10.0 kPa	n/a	64.0%	n/a	n/a	86.0%	n/a	n/a	n/a	n/a
Cassinotto et al., 2021 [25]	Retrospective	France	577											
				NFS	n/a	n/a	n/a	n/a	n/a	n/a	n/a	0.700	0.700	0.730
				FIB-4	n/a	n/a	n/a	n/a	n/a	n/a	n/a	0.700	0.740	0.800
				VCTE	n/a	n/a	n/a	n/a	n/a	n/a	n/a	0.800	0.820	0.850
				SWE	n/a	n/a	n/a	n/a	n/a	n/a	n/a	0.840	0.880	0.860
				FIB-4/SWE	n/a	n/a	71.4%	n/a	n/a	91.4%	n/a	n/a	0.837	n/a
				FIB-4/VCTE	n/a	n/a	66.0%	n/a	n/a	91.5%	n/a	n/a	0.814	n/a
				FIB-4/SWE/VCTE	n/a	n/a	71.5%	n/a	n/a	87.9%	n/a	n/a	0.811	n/a
				FIB-4/VCTE/SWE	n/a	n/a	69.7%	n/a	n/a	89.5%	n/a	n/a	0.813	n/a
Newsome et al., 2020 [14]	Prospective	Multinational	981											
				FIB-4	>3.25 and <1.30	7.0%	n/a	n/a	76.0%	n/a	n/a	0.740	n/a	n/a
				NFS	>0.676 and <-1.455	19.0%	n/a	n/a	52.0%	n/a	n/a	0.680	n/a	n/a
				FAST	>0.67 and <0.35	49.0%	n/a	n/a	64.0%	n/a	n/a	0.850	n/a	n/a
Anstee et al., 2019 [26]	Retrospective	Multinational	3202											
				FIB-4	>2.67 and <1.3	n/a	82.0%	n/a	n/a	93.0%	n/a	n/a	0.780	n/a
				NFS	>0.676 and <-1.455	n/a	89.0%	n/a	n/a	89.0%	n/a	n/a	0.740	n/a
				ELF	>11.3 and <9.8	n/a	74.0%	n/a	n/a	98.0%	n/a	n/a	0.800	n/a
				VCTE	>11.4 and <9.9 kPa	n/a	83.0%	n/a	n/a	71.0%	n/a	n/a	0.800	n/a
				FIB-4 & ELF	>2.67 and <1.3/>11.3 and <9.8	n/a	89.0%	n/a	n/a	99.0%	n/a	n/a	n/a	n/a
				FIB-4 & VCTE	>2.67 and <1.3/>11.4 and <9.9 kPa	n/a	94.0%	n/a	n/a	97.0%	n/a	n/a	n/a	n/a
				NFS & ELF	>0.676 and <-1.455/>11.3 and <9.8	n/a	94.0%	n/a	n/a	99.0%	n/a	n/a	n/a	n/a
				NFS & VCTE	>0.676 and <-1.455/>11.4 and <9.9 kPa	n/a	96.0%	n/a	n/a	97.0%	n/a	n/a	n/a	n/a
				FIB-4/ELF	>2.67 and <1.3/>11.3 and <9.8	n/a	69.0%	n/a	n/a	92.0%	n/a	n/a	n/a	n/a
				FIB-4/VCTE	>2.67 and <1.3/>11.4 and <9.9 kPa	n/a	77.0%	n/a	n/a	89.0%	n/a	n/a	n/a	n/a
Boursier et al., 2019 [27]	Prospective	France	938											
				FIB-4	n/a	n/a	91.9%	n/a	n/a	96.3%	n/a	0.711	0.763	0.784
				NFS	n/a	n/a	89.5%	n/a	n/a	94.2%	n/a	0.712	0.722	0.749
				FibroTest	n/a	n/a	n/a	n/a	n/a	n/a	n/a	0.760	0.738	0.768
				Hepascore	n/a	n/a	n/a	n/a	n/a	n/a	n/a	0.712	0.756	0.798
				FibroMeter	n/a	n/a	88.7%	n/a	n/a	93.7%	n/a	0.751	0.793	0.815
				VCTE	n/a	n/a	90.3%	n/a	n/a	97.4%	n/a	0.826	0.840	0.872
				FibroMeter & VCTE	n/a	n/a	90.3%	n/a	n/a	91.5%	n/a	0.833	0.866	0.897
				FIB-4/VCTE	n/a	n/a	84.7%	n/a	n/a	94.7%	n/a	n/a	n/a	n/a
				NFS/VCTE	n/a	n/a	83.1%	n/a	n/a	92.1%	n/a	n/a	n/a	n/a
				FibroMeter/VCTE	n/a	n/a	83.1%	n/a	n/a	92.1%	n/a	n/a	n/a	n/a
				NFS/FibroMeter & VCTE	n/a	n/a	83.1%	n/a	n/a	87.3%	n/a	n/a	n/a	n/a
				FIB-4/FibroMeter & VCTE	n/a	n/a	86.3%	n/a	n/a	90.5%	n/a	n/a	n/a	n/a
				FibroMeter/FibroMeter & VCTE	n/a	n/a	84.7%	n/a	n/a	88.9%	n/a	n/a	n/a	n/a
				VCTE/FibroMeter & VCTE	n/a	n/a	85.5%	n/a	n/a	92.6%	n/a	n/a	n/a	n/a

^a^ = based on calculated algorithm.

“/" indicates sequential testing with the initial and subsequent test listed before and after the "/", respectively.

### 3.3 NAFLD Fibrosis Score

The NAFLD Fibrosis Score [NFS] incorporates age, body mass index [BMI], the presence of diabetes mellitus or impaired fasting glucose, platelet count, albumin, ALT, and AST. The formula is as follows: −1.675 + (0.037 x age) + (0.094 x BMI) + (1.13 if diabetes present) + (0.99 x (AST/ALT)) – (0.013 x platelets) – (0.66 x albumin). NFS does not require a special laboratory and can be easily calculated by the practitioner utilizing readily available on-line calculators.

Tapper et al. validated the NFS in a study of 733 biopsy-proven patients with NAFLD [19]. Across the derivation and validation cohorts, implementing a lower NFS threshold of less than −1.455 demonstrated a NPV of 93% and 88% for the detection of advanced fibrosis, respectively. Meanwhile, instituting a higher NFS threshold of greater than 0.676 revealed a 90% and 82% PPV across the same cohorts. Utilization of this dual threshold would have averted 75% of liver biopsies performed in the patient population.

A meta-analysis of 12,604 biopsy-proven NAFLD patients compared FIB-4 and NFS testing with respect to diagnostic accuracy for advanced fibrosis [20]. Using the lower thresholds of 1.3 for FIB-4 and -1.455 for NFS, these noninvasive tests were found to have 76% sensitivity and 67% specificity in addition to 81% sensitivity and 64% specificity, respectively. However, using the higher threshold of 2.67 for FIB-4 demonstrated 39% sensitivity and 95% specificity, while the 0.676 threshold for NFS was associated with 34% sensitivity and 94% specificity for advanced fibrosis. This meta-analysis went on to investigate these noninvasive tests with dual thresholds, sometimes referred to as “the grey zone” in the literature. Using dual thresholds for FIB-4 [1.3 and 2.67], sensitivity was found to be 65% and specificity 93%. NFS dual thresholds of −1.455 and 0.676 raised sensitivity and specificity to 61% and 93%, respectively. However, significant heterogeneity was noted across all outcomes. Overall, this meta-analysis highlights the demand for optimized testing limits. The dual threshold approach appears to demonstrate through its higher specificity that FIB-4 has slight superiority of detecting the presence of advanced fibrosis among NAFLD patients, whereas NFS is better at ruling out advanced fibrosis based on its sensitivity.

Limitations of NFS have been documented among obese patients. One study analyzed the role of noninvasive fibrosis assessment among 386 biopsy-proven NAFLD patients both with and without obesity [21]. Five noninvasive tests were subsequently analyzed among patients with and without obesity. AUROC for noninvasive testing between patients with and without obesity were found to be 0.698 versus 0.812 for AST/ALT, 0.760 versus 0.833 for BARD score, 0.845 versus 0.726 for APRI, 0.887 versus 0.871 for FIB-4, and 0.873 versus 0.868 for NFS, respectively, in the detection of advanced fibrosis. Overall, FIB-4 demonstrated stable diagnostic accuracy across all stages of obesity, while NFS appeared to overestimate fibrosis stage among patients with morbid obesity. However, when adjusting the NFS for patients with BMI >40, this improved the AUROC for NFS to 0.838.

The FLINT trial was a randomized controlled trial analyzing the impact of 72 weeks of obeticholic acid versus placebo among patients with NASH. A post-hoc analysis of this trial reveals that reduction in levels of APRI and FIB-4 scores were significantly associated with a minimum one-stage improvement in histologic fibrosis with p values of 0.015 and 0.036, respectively [22]. However, no such correlation was found with NFS scoring [p = 0.201].

Overall, NFS has proved one of the most robust noninvasive tools to date. The most widely utilized thresholds of −1.455 and 0.676 have served as an accurate surrogate marker for the detection of fibrosis among patients with NALFD [Table 2, Table 3, Table 4, Table 5, Table 6]. Using a lower limit of −1.455 carries a sensitivity of 81% while an upper threshold on 0.676 increases the specificity to 94%.

### 3.4 Hepamet Fibrosis Score

The Hepamet Fibrosis Score [HFS] is based on age, gender, diabetes mellitus, platelets, albumin, AST, and HOMA [homeostatic model assessment for insulin resistance]. A multicenter European cross-sectional study followed by a longitudinal assessment of long term outcomes was performed among 1,173 biopsy proven NALFD patients comparing APRI, BARD, FIB-4, NFS, and HFS to liver biopsy [23]. HFS demonstrated superior diagnostic accuracy [AUROC 0.758 and 0.805 for significant and advanced fibrosis, respectively] as compared to both FIB-4 [AUROC 0.697 and 0.733] and NFS [AUROC 0.700 and 0.761], however all three tests were superior to APRI and BARD scores. NFS and FIB-4 were superior in the detection of histologic cirrhosis. Furthermore, FIB-4 and NFS were the best tests in the longitudinal follow up and prediction of liver-related events.

NFS does not require a special send out and can be easily calculated by the practitioner utilizing readily available on-line calculators.

## 4 Noninvasive serum biomarkers targeting collagen turnover and extracellular matrix remodeling

Several novel biomarkers have developed that identify collagen turnover, extracellular matrix deposition, and fibrogenesis. Common targets include alpha-2 macroglobulin, hyaluronic acid, type III procollagen, and tissue inhibitor of metallopeptidase-1 [TIMP1]. Comparison of many individual tests and laboratory panels are listed in Table 4. Common thresholds for advanced fibrosis are listed in Table 2. One retrospective analysis validated a combined algorithm combining the alpha-2 macroglobulin, hyaluronic acid, and TIMP1 testing across 396 biopsy-proven NAFLD patients [24]. Combining all three of these tests was shown to be superior in the diagnostic accuracy for the detection of advanced fibrosis, as compared to each individual test alone, in addition to analysis versus either FIB-4 or NFS testing. Additionally, TIMP1 outperformed multiple other serum biomarkers as a significant, independent predictor of advanced fibrosis when compared to biopsy-proven NAFLD [25].

### 4.1 Enhanced liver fibrosis test

The Enhanced Liver Fibrosis [ELF] test is comprised of extracellular matrix targets and markers of collagen turnover, which includes hyaluronic acid, amino-terminal propeptide of type III procollagen, and TIMP1. ELF requires the specimen to be sent to a specialized laboratory.

ELF has been validated in the detection of fibrosis among 192 biopsy-proven NAFLD patients [26]. ELF was compared to “simple panels,” or in this case, NFS testing. Reporting AUROC, NFS was found to have 0.860 for significant fibrosis and 0.890 for advanced fibrosis, whereas ELF demonstrated 0.90 for significant fibrosis and 0.93 for advanced fibrosis. Moreover, when NFS and ELF were combined together, the AUROC for significant fibrosis was 0.93 and advanced fibrosis was 0.98. This benchmark study established ELF as a robust noninvasive test for the detection of fibrosis among patients with NAFLD.

Also, as opposed to one of the shortcomings reported for NFS testing, the results of ELF testing do not appear to fluctuate based on obesity. One randomized study has demonstrated that moderate weight loss did not affect the ELF score significantly [27].

### 4.2 FibroTest®

FibroTest®, or in the USA referred to as FibroSure®, is a noninvasive test consisting of five biomarkers: bilirubin, gamma-glutamyl transferase, haptoglobin, apolipoprotein A1, and alpha-2 macroglobulin. This noninvasive test was first described in hepatitis C [28], and subsequent studies among patients with other forms of chronic liver disease including NAFLD. FibroTest ®, requires the specimen to be sent to a specialized laboratory. In a large meta-analysis including 267 biopsy-proven NAFLD patients, the pooled AUROC was calculated at 0.840 in the detection of significant fibrosis by using the FibroTest® [29].

### 4.3 ADAPT

One study details the ADAPT score, which includes age, history of type 2 diabetes mellitus, PRO-C3 [N-terminal type III collagen propeptide], and platelet count, among 431 biopsy-proven NAFLD patients as analyzed the association with advanced fibrosis [30]. Using a threshold of greater than 6.3287, the ADAPT score was found to have AUROC 0.86, sensitivity 90.9%, and specificity 72.7% for advanced fibrosis. ADAPT was found to be statistically superior in the assessment of fibrosis as compared to other noninvasive biomarkers including APRI, FIB-4, and NFS. Furthermore, PRO-C3 levels were found to be significantly elevated among patients with advanced fibrosis with AUROC 0.81 for advanced fibrosis.

ADAPT does not require a special laboratory and can be easily calculated by the practitioner utilizing readily available on-line calculators.

### 4.4 FibroMeter®

FibroMeter*®* provides a panel of noninvasive biomarkers combining both conventional-based testing with markers of collagen and extracellular matrix remodeling. The FibroMeter V2G® testing utilizes age, gender, BUN, platelet count, INR, AST, alpha-2 macroglobulin, and hyaluronic acid. FibroMeter V3G® substitutes gamma-glutamyl transferase for hyaluronic acid while utilizing all the other FibroMeter V2G® components. FibroMeter®requires the specimen to be sent to a specialized laboratory. FibroMeter® scores reported on a scale of 0–1, with a score of 1 being associated with more advanced fibrosis.

A prospective, biopsy-controlled Austrian cohort study compared liver biopsy for patients with suspected NAFLD to six noninvasive tests, including NFS, FIB-4, ELF®, FibroMeter[V2G]®, FibroMeter[V3G]®, and VCTE [31]. For endpoints of significant fibrosis [F ≥ 2], advanced fibrosis [F ≥ 3], and advanced fibrosis plus NASH, respectively, analysis reveals more accurate diagnosis made by using the ELF score [AUROC 0.85, 0.90, 0.90], FibroMeterV2G [AUROC 0.86, 0.88, 0.89], FibroMeterV3G [AUROC 0.84, 0.88, 0.88], and VCTE [AUROC 0.87, 0.95, 0.91] as compared to FIB-4 [AUROC 0.80, 0.82, 0.81] or NFS [AUROC 0.78, 0.80, 0.79]. Another large-scale meta-analysis found no statistically significant differences between diagnostic accuracy of the two different FibroMeter ® versions [32].

### 4.5 Hepascore®

The Hepascore®, developed in Australia, includes components of age, gender, bilirubin, gamma-glutamyl transferase, hyaluronic acid, and alpha-2 macroglobulin. Hepascore® requires the specimen to be sent to a specialized laboratory. Scores are reported on a scale of 0–1, with scores closer to 1 being associated with advanced fibrosis.

One of the initial studies analyzing Hepascore® was performed to accurately diagnose liver fibrosis in chronic hepatitis C [33]. One recent study compared Hepascore® with multiple other tests including NFS, FIB-4, APRI, FibroTest®, FibroMeter®, BARD score, and VCTE [34]. The primary aim of this study was to assess the diagnostic accuracy of these noninvasive tests for advanced fibrosis as compared to histology among 454 biopsy-proven NAFLD patients. Overall, VCTE and FibroMeter® [V2G] demonstrated superior AUROC with 0.831 and 0.817, respectively, with Hepascore® performing as the next best test with AUROC 0.778 for advanced fibrosis. These noninvasive tests had a similar hierarchy upon assessment for cirrhosis-based comparison of liver histology.

### 4.6 NIS4

A novel biomarker panel known as NIS4, comprised of miR-34a-5p, alpha-2 macroglobulin, YKL-40, and glycated hemoglobin, was recently validated among a prospective derivation and validation cohort in the detection of NASH and advanced fibrosis [35]. Among 239 biopsy-proven NAFLD patients, the AUROC for NIS4 was calculated to be 0.80. Values less than 0.36 accurately ruled out NASH and advanced fibrosis with 81.5% sensitivity, 63% specificity, and NPV 77.9%. A NIS4 threshold greater than 0.63 revealed 87.1% specificity, 50.7% sensitivity, and PPV 79.2% for the diagnosis of NASH and advanced fibrosis.

## 5 Noninvasive imaging modalities

### 5.1 Vibration controlled transient elastography

Vibration controlled transient elastography [VCTE] is a noninvasive imaging modality that calculates the liver stiffness measurement [LSM] through the propagation of mechanical vibration through hepatic tissue. VCTE has become a widely utilized modality, most commonly via FibroScan® or FibroTouch®. Prospective analysis from over 400 patients in the United Kingdom compared VCTE vs liver biopsy among NAFLD patients [36]. VCTE identified patients with fibrosis with AUROCs of 0.77 [95% CI 0.72–0.82] for significant fibrosis, 0.80 [95% CI 0.75–0.84] for advanced fibrosis, and 0.89 [95% CI 0.84–0.93] for cirrhosis. Another study prospectively followed 393 biopsy-proven NAFLD patients [37]. It was found that VCTE had diagnostic accuracy with AUROC of 0.83 and 0.93 for advanced fibrosis and cirrhosis, respectively.

A prospective study among 237 Chinese patients compared liver biopsy with VCTE [38]. There were a number of patients with concomitant hepatitis B in this study, which were excluded. Overall, VCTE produced an AUROC of 0.710, 0.710, and 0.770 for significant fibrosis, advanced fibrosis, and cirrhosis, respectively. Utilizing this noninvasive test, VCTE with FibroTouch demonstrated a sensitivity of 58%, 68%, and 80% in addition to specificity of 82%, 72%, and 71% for significant fibrosis, advanced fibrosis, and cirrhosis, respectively. Similar results have been corroborated across previous studies analyzing the diagnostic accuracy of VCTE among NAFLD patients, the results of which are detailed in Table 5 [34,[39], [40], [41], [42], [43], [44], [45], [46]].

Despite its readily available and widespread use, VCTE does harbor some significant limitations. VCTE can also lead to inaccurate testing in the presence of ascites, large pleural effusions, or congestive hepatopathy. Secondly, LSM values and sensitivity are altered for testing among morbidly obese patients, which is clearly a common risk factor among NAFLD.

Some of these limitations can be circumvented with the various probe types. The S1 and S2 probes are used primarily among patients less than eighteen years of age. The determination of S1 versus S2 is dictated by the thoracic parameter measurement [47]. Among adult patients, both the M and XL probes are used. The M probe is the primary probe and generally utilized first. However, the M probe has been demonstrated to have a high failure rate in the detection and calculation of LSM among obese patients [48]. The development of the XL probe has been shown to reduce VCTE failure rates among patients with obesity and more accurately calculate LSM [49].

### 5.2 Shear wave elastography

Shear wave elastography [SWE] is a similar noninvasive technique to VCTE, however SWE utilizes a two-dimensional ultrasound probe to generate a focused acoustic beam which propagates through hepatic tissue and measures LSM. The majority of evidence for SWE has been performed among patients with viral hepatitis, and in this setting, SWE appears to portend similar diagnostic accuracy for fibrosis as compared to VCTE [50]. However, validation of SWE in the assessment of fibrosis among patients with NAFLD need to be a focus of future clinical trials. There is a current paucity of head-to-head comparisons, but studies including SWE across combination or multiple noninvasive modalities will be discussed below.

### 5.3 Fibroscan-AST™ (FAST™) score

One prospective, multicenter study investigated the utility of the FAST*™* score, which combines VCTE and CAP with FibroScan in addition to AST, in the detection of significant fibrosis among a derivation cohort and a 981 patient validation cohort among patients with biopsy-proven NASH [51]. In the detection of significant fibrosis, the FAST*™* score outperformed NFS and FIB-4 with AUROC of 0.85, 0.68, and 0.74, respectively. A relatively simple combination of FibroScan*™* plus AST may provide improved clinical utility in the detection of NAFLD patients with significant fibrosis, although this study was restriction to patients with NASH.

### 5.4 Velacur™

Velacur™ is another novel noninvasive imaging modality developed in the fibrosis assessment among patients with NAFLD. It utilizes multiple, steady-state ultrasound waves in order to generate a three-dimensional liver tissue sample, including noninvasive calculations for hepatic fibrosis and steatosis [52]. Velacur™ still needs to be validated across larger, clinical trials, however appears to be an attractive noninvasive fibrosis assessment in the future management for patients with NAFLD.

### 5.5 Magnetic Resonance Elastography (MRE)

**Fig. 1:**
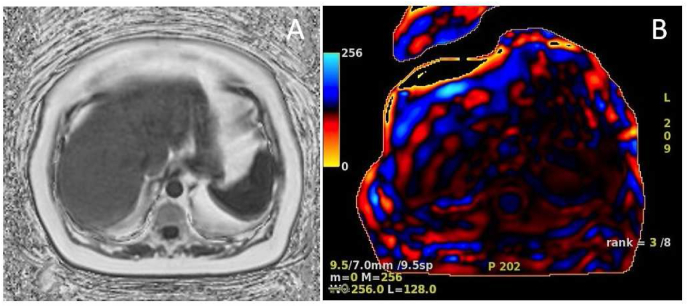
**Magnetic Resonance Elastography (MRE)**. The following MRE images are taken from a patient with history of nonalcoholic fatty liver disease. This patient has normal liver morphology and a 12% hepatic fat fraction [Fig. 1A]. Color wave images noted in Fig. 1B with stage 3–4 fibrosis noted with hepatic stiffness 4.4 kPa.

MRE uses magnetic resonance imaging and low frequency mechanical waves to non-invasively measure the stiffness of liver tissue. An example of the qualitative and quantitative data available with MRE is detailed in Fig. 1. While this method can give an accurate and holistic assessment of hepatic fibrosis, it is not without limitations. MRE carries significantly increased cost and resources associated with this imaging modality. Furthermore, not all medical centers are equipped with MRE, making this a testing strategy of limited availability.

One study from the Netherlands prospectively compared MRE and VCTE via FibroScan among thirty-seven biopsy-proven NAFLD patients [53]. As compared to fibrosis on liver biopsy for the diagnosis of advanced fibrosis, AUROC for MRE and VCTE was found to be 0.920 and 0.770, respectively. MRE was found to have 100% sensitivity and 78.6% specificity, while VCTE had 87.5% sensitivity and 69% specificity. This was a small, yet compelling, clinical study, comparing MRE and VCTE with liver biopsy in the assessment of advanced fibrosis in NAFLD.

A meta-analysis compared several noninvasive serum tests and imaging modalities in the assessment of fibrosis in 13,046 patients with NAFLD as compared to liver biopsy [54]. For the diagnostic accuracy of significant fibrosis, the mean AUROC was found to be 0.70 for APRI, 0.64 for BARD score, 0.75 for FIB-4, 0.72 for NFS, 0.83 for VCTE [M probe], 0.82 for VCTE [XL probe], 0.89 for SWE, and 0.88 for MRE. The mean AUROC was found to be 0.75 for APRI, 0.73 for BARD score, 0.80 for FIB-4, 0.78 for NFS, 0.87 for VCTE [M probe], 0.86 for VCTE [XL probe], 0.91 for shear wave elastography (SWE), and 0.93 for MRE with respect to advance fibrosis assessment. Finally, the mean AUROC for accurately detecting cirrhosis were 0.75 for APRI, 0.70 for BARD score, 0.85 for FIB-4, 0.83 for NFS, 0.92 for VCTE [M probe], 0.94 for VCTE [XL probe], 0.97 for SWE, and 0.92 for MRE. In the pooled analyses, there was significant heterogeneity noted in APRI [I^2^ = 84%]and FIB-4 [I^2^ = 79%] for the assessment of significant fibrosis as well as MRE [I^2^ = 51%] in the evaluation of advanced fibrosis. While these noninvasive serum tests appear to have robust NPV for advanced fibrosis, they appear to have relatively high false positive rates thus hindering their diagnostic yield. Overall, SWE and MRE appear to provide the highest diagnostic accuracy in the assessment of fibrosis among patients with NAFLD, with FIB-4 and NFS the superior noninvasive blood tests found in this meta-analysis.

## 6 Combination, sequential, and algorithmic testing

Several clinical studies have attempted to combine or sequentially perform noninvasive serum biomarkers with one another or in tandem with an imaging modality. Some of these high impact studies are categorized in Table 6.

Post-hoc analysis of screening data from STELLAR-3 and STELLAR-4 trials compared histology from liver biopsy to individual, combination, and stepwise testing of FIB-4, NFS, ELF, and VCTE among 3202 patients [55]. The primary outcome was the detection of advanced fibrosis. ELF and VCTE demonstrated equal diagnostic accuracy, both with AUROC of 0.800, while FIB-4 and NFS achieved AUROC of 0.780 and 0.740, respectively. Combination testing with either FIB-4 and VCTE, NFS and ELF, or NFS and VCTE demonstrated robust findings with both sensitivity and specificity ≥94%. Sequential testing using either FIB-4 then ELF or FIB-4 followed by VCTE, was proven to have lower sensitivity and similar specificity. Overall, individual noninvasive testing or in combination can help distinguish advanced fibrosis and potentially obviate the need for liver biopsy.

One large study including 938 French patients with biopsy-proven NAFLD randomized patients into derivation and validation sets in which NFS, FIB-4, FibroTest,® Hepascore®, FibroMeter®, and VCTE was compared to liver biopsy [56]. An additional arm, FibroMeter^VCTE^®, combined the FibroMeter® serum testing with VCTE. 90% sensitivity and specificity for each test were the designed thresholds for advanced fibrosis in the derivation set. Of serum tests, FibroMeter® had the highest AUROC for fibrosis while VCTE demonstrated superior accuracy than any serum noninvasive testing. 90% sensitivity in the derivation set using thresholds of NFS -1.669, FIB-4 1.04, FibroMeter® 0.26, VCTE 8.0 kPa, and FM^VCTE^ 0.32 ruled out advanced fibrosis with NPV 85–90%. Subsequently, new proposed algorithms in the validation set demonstrate that sequential, second-line testing with FIB-4-FM^VCTE^ and VCTE-FM^VCTE^ achieved superior diagnostic accuracy for advanced fibrosis. FIB-4 followed by FM^VCTE^ revealed diagnostic accuracy 88.8%, sensitivity 86.3%, specificity 90.5%, NPV 91.0%, PPV 85.6% for advanced fibrosis as compared to liver biopsy. Meanwhile, VCTE followed by FM^VCTE^ illustrated diagnostic accuracy of 89.8%, sensitivity 85.5%, specificity 92.6%, NPV 90.7%, PPV 88.3%. Patients who underwent second-line testing with FIB-4-FM^VCTE^ and VCTE-FM^VCTE^ required liver biopsy in only 21.1% and 22.0% of cases, respectively, as opposed to 63.6% who underwent single NFS testing. Overall, this unique, randomized study establishes the clinical utility of second-line testing across this validated algorithm.

A French retrospective study among 577 biopsy-proven NAFLD patients examined the role of FIB-4, VCTE, and shear wave elastography in assessing fibrosis [57]. This study primarily looked at outcomes of advanced fibrosis and number of patients requiring liver biopsy following the results of each test. The diagnostic accuracy for each individual test was expressed in AUROC as detailed in Table 6. Escalating to a two-step method vastly improved accuracy compared to blood testing alone, as seen with AUROC 0.837 for FIB-4/SWE and AUROC 0.814 for FIB-4/VCTE. While no significant differences were found in the diagnostic accuracy between these two groups, there was a significantly greater percentage of patients requiring liver biopsy following the results of their tests in the FIB-4/SWE group [24.6%] as compared to those who underwent FIB-4/VCTE [15.3%; p < 0.001]. Taking one step further to a three-step method of testing, there was no significant difference between FIB-4/VCTE/SWE versus a FIB-4/SWE/VCTE strategy, however using this three-step method markedly decreased the need for liver biopsy for patients in the “grey zone,” whose first testing produced an indeterminant 8–10 kPa quantification of fibrosis.

A large meta-analysis including pooled patient data from 37 clinical studies across sixteen countries and 5735 biopsy-proven NAFLD patients evaluated the sensitivity and specificity of various non-invasive markers of fibrosis in assessing advanced fibrosis [58]. Diagnostic accuracy for individual AST/ALT, APRI, FIB-4, NFS, and VCTE noninvasive testing was first calculated and found to have pooled AUROC of 0.640, 0.700, 0.760, 0.730, and 0.850, respectively. The authors conducted sequential algorithmic testing, combining either FIB-4 or NFS with VCTE at various cut-off, or threshold, values. One common clinical practice is FIB-4 [with cut-offs <1.3 and ≥ 2.67] followed by VCTE [<8.0 kPa and ≥ 10.0 kPa]. This study found a 66% sensitivity and 86% specificity, with a 9% false negative rate and approximately 33% of patients still requiring biopsy when using this algorithm to assess fibrosis. The remainder of these sensitivities and specificities for these tested algorithms are detailed in Table 6. The authors concluded that sequential testing of FIB-4 with lower cut-off of less than 1.3 followed by VCTE less than 8.0 kPa effectively ruled out advanced fibrosis. Similarly, by raising the upper limit threshold, the diagnosis of cirrhosis can be made, and thus obviate the need for liver biopsy, with 95% specificity using FIB-4 cut-off of ≥ 3.48 and VCTE ≥ 20.0 kPa.

## 7 Future directions

Better and more accurate non-invasive testing to assess the degree of hepatic fibrosis are needed. Several new methods for testing are emerging for the assessment of fibrosis in NAFLD. One of the earlier metabolomic studies illustrated that levels of sulfated dehydroepiandrosterone sulfate [DHEA-S] were decreased among patients with NAFLD and there was a dose-dependent association between decreasing DHEA-S level and increasing stage of fibrosis [59]. The mean AUROC for DHEA-S was calculated at 0.830 for the presence of advanced fibrosis. Another clinical study set forth to investigate the diagnostic implementation for metabolomic testing among 156 biopsy-proven NAFLD patients [60]. This study used a proprietary metabolite test known as Metabolon®, which following a derivation cohort, targeted ten metabolites including eight lipids [5-alpha-androstan-3-beta monosulfate, pregnanediol-3-glucuronide, androsterone sulfate, epiandrosterone sulfate, palmitoleate, dehydroisoandrosterone sulfate, 5-alphaandrostan-3-beta disulfate, glycocholate], one amino acid [taurine], and one carbohydrate [fucose]. Among this cohort, metabolite testing demonstrated significantly superior diagnostic accuracy for advanced fibrosis as compared to FIB-4 and NFS with AUROC reported at 0.940, 0.840, and 0.780, respectively. However, the external validity of this study remains limited due to the nature of its cross-sectional study design and overall lack of availability and resources for routine metabolomic testing among most patients with NAFLD.

Genetic testing may have a role in the assessment of hepatic fibrosis in NAFLD patients. The presence of the patatin-like phospholipase domain-containing protein 3 [PNPLA3] has been associated with NAFLD. NAFLD Patients with the specific PNPLA3 rs738409 single nucleotide polymorphism have been demonstrated to be at high risk for hepatic decompensation, liver-related events, and liver related mortality [61]. Among a cohort of 772 biopsy-proven NAFLD patients, the presence of PNPLA3 rs738409 was associated with progression of fibrosis [62]. The diagnostic accuracy of advanced fibrosis was reported as AUROC 0.871 for FIB-4 and AUROC 0.887 with VCTE testing. Using an alternative novel modality via transcriptomics testing, another study assessed the role of hepatocyte interleukin-32 [IL-32] among patients with and without PNPLA3 rs738409 [63]. The authors concluded that when IL-32 is overexpressed, independent of PNPLA3 genotype, it significantly correlated with increased hepatic steatosis and significant fibrosis. However, some current limitations among patients with varying PNPLA3 genotypes may exist. A retrospective study among 58 controls and 349 patients with biopsy-proven NAFLD explored the diagnostic accuracy of noninvasive testing stratified by PNPLA3 genotype [64]. Overall, there were vast differences among BARD score [AUROC 0.805 and 0.532] and FIB-4 testing [AUROC 0.662 and 0.801] in the detection of significant fibrosis among patients with or without PNPLA3 rs738409, respectively. The incorporation of genetic testing may be a direction of future emphasize for noninvasive testing, however current limitations exist and will need to be further elucidated in future clinical trials.

There has been a recent flurry of preliminary data regarding genetic, epigenetic, transcriptomics, metabolomic, and proteomic testing in the fibrosis assessment among patients with NAFLD. However, these specific noninvasive tests remain limited given their expense and availability. They must be further substantiated with reproducible findings across multiple trials. These novel noninvasive tests will be important to not only risk stratify but also diagnose fibrosis in NAFLD after being targeted by future studies.

## 8 Conclusion

Currently, routine screening for NAFLD is not recommended, even among high risk groups, based on most recent AASLD guidelines given lack of evidence and long term cost-effectiveness of screening [65]. However, the AASLD does recommend the routine utilization of noninvasive testing in patients with high index of suspicion, with NFS, FIB-4, and/or VCTE as first tests of choice at the time the most recent guideline regarding NAFLD was published [65].

NAFLD is and will continue to be a global problem. Due to its high prevalence and the lack of feasibility to utilize liver biopsy regularly, non-invasive assessment of hepatic fibrosis in NAFLD is needed to determine prognosis, progression, and potential need for therapy, once available. Over the past decade, there have been numerous breakthroughs in the development of noninvasive tests in the assessment of fibrosis among patients with NAFLD. While liver biopsy is still necessary in a subset of patients, the growing role and accuracy of noninvasive tests can potentially obviate the need for the extra resources and risk of liver biopsy, which is imperative given the exponential increase in NAFLD prevalence.

NFS and FIB-4 currently serve as the benchmark for fibrosis assessment in patients with NAFLD. This is based on their diagnostic accuracy and implementation of widely utilized, easily accessible laboratory parameters. The utilization of dual thresholds as well as noninvasive serum biomarkers being used in tandem with imaging modalities has demonstrated to even further heighten the diagnostic accuracy of fibrosis. This strategy has recently been highlighted by expert opinion from the American College of Gastroenterology [66] and the European Association for the Study of the Liver [67].

Other emerging and future noninvasive tests must be constantly reassessed for their efficacy of detecting fibrosis among this patient population, ideally in direct comparison to NFS and FIB-4. Several noninvasive panels have already been well-established, such as ELF. Alternative emerging testing strategies using HFS, FibroMeter, Hepascore, and NIS4 also appear to be attractive targets. The development of new noninvasive testing based on genetic, epigenetic, and transcriptomic modalities is ongoing. This testing modality could ultimately lead to a highly patient-specific approach in the management of NAFLD, not only tailored to an individual's phenotype, but also targeting the underlying genetic targets responsible for its pathophysiology. All of these emerging noninvasive tests should serve as an aim for future clinical studies.

Overall, noninvasive testing for the assessment of fibrosis is crucial, especially for a patient-centered approach that caters to avoidance of unnecessary liver biopsies. Noninvasive testing will take on a larger clinical role as the incidence of NAFLD continues to rise. Noninvasive fibrosis assessment will remain a stratification tool for patients with NAFLD for years to come.

## CRediT authorship contribution statement

**David Bernstein:** Conceptualization, Methodology, Validation, Formal analysis, Writing – original draft, Writing – review & editing, Supervision, Project administration. **Alexander J. Kovalic:** Conceptualization, Methodology, Validation, Formal analysis, Writing – original draft, Writing – review & editing.

## Declaration of competing interest

The authors declare that they have no known competing financial interests or personal relationships that could have appeared to influence the work reported in this paper.
